# Epidemiological characteristics and post-exposure prophylaxis of human rabies in Chongqing, China, 2007–2016

**DOI:** 10.1186/s12879-017-2830-x

**Published:** 2018-01-03

**Authors:** Li Qi, Kun Su, Tao Shen, Wenge Tang, Bangzhong Xiao, Jiang Long, Han Zhao, Xi Chen, Yu Xia, Yu Xiong, Dayong Xiao, Liangui Feng, Qin Li

**Affiliations:** 1 Chongqing Municipal Center for Disease Control and Prevention, No.8, Changjiang 2nd Road, Yuzhong District, Chongqing, China; 2 Chinese Field Epidemiology Training Program, Chinese Center for Disease Control and Prevention, Beijing, China; 3 Department of Military Epidemiology, College of Military Prevention, Third Military Medical University, Chongqing, China; 4 Chongqing Medical and Pharmaceutical College, Chongqing, China

**Keywords:** Human rabies, Post-exposure prophylaxis, Epidemiology

## Abstract

**Background:**

According to the global framework of eliminating human rabies, China is responding to achieve the target of zero human death from dog-mediated rabies by 2030. Chongqing is the largest municipality directly under central government in China. We described the epidemiological characteristics and post-exposure prophylaxis (PEP) of human rabies in this area, in order to provide a reliable epidemiology basis for further control and prevention of human rabies.

**Methods:**

The most updated epidemiological data for human rabies cases from 2007 to 2016 in Chongqing were collected from the National Disease Reporting Information System. A standardized questionnaire was applied to the human rabies cases or family members of cases as proxy to investigate the PEP situation.

**Results:**

A total of 809 fatal human rabies cases were reported in Chongqing from 2007 to 2016. There was a trend of gradual annual decline about number of cases from 2007 to 2013, followed by stable levels until 2016. Rabies was mostly reported in summer and autumn; a majority of cases were noted in farmers (71.8%), especially in males (65.3%). The cases aged 35–74 and 5–14 years old accounted for 83.8% of all the cases. We collected information of 548 human rabies cases’ rabies exposure and PEP situation. Of those, 95.8% of human rabies cases were victims of dog bites or scratch, and 53.3% of these dogs were identified as stray dogs. Only 4.0% of the domestic dogs were reported to have been vaccinated previously. After exposure, 87.8% of the 548 human rabies cases did not seek any medical services. Further investigation showed that none of the 548 cases received timely and properly standardized PEP.

**Conclusion:**

Human rabies remains a major public health problem in Chongqing, China. Dogs are the main reservoir and source of human rabies infection. Unsuccessful control of canine rabies and inadequate PEP of cases might be the main factors leading to the serious human rabies epidemic in this area. An integrated “One Health” approach should be encouraged and strengthened in this area; with combined effort it would be possible to achieve the elimination of human rabies in the expected date.

**Electronic supplementary material:**

The online version of this article (10.1186/s12879-017-2830-x) contains supplementary material, which is available to authorized users.

## Background

Rabies is a neglected zoonosis infectious disease and the most lethal infectious disease with fatality rate in humans approaching 100% [1]. The number of human rabies deaths was estimated to be 61,000 globally [2], corresponding to over 3.7 million disability-adjusted life-years and 8.6 billion USD economic losses every year [3]. China is one of the countries that experience the most serious impact from this disease, with human rabies cases ranking second in the world, after India [4, 5]. The 2015 Chinese yearbook of health statistics showed that rabies death ranked the third in the list of 39 notifiable infectious diseases, following AIDS and tuberculosis [6]. The disease was severely underreported because most victims died at home, which led to an insufficient prioritization of rabies prevention in public health agendas [7]. Chongqing is the largest municipality under direct control of the national government, located in the Southwestern region of China, sharing borders with Hunan Province and Guizhou Province, which are two of the most seriously affected provinces by human rabies in China [8].

According to the global framework of eliminating rabies, China is responding to the target of zero human rabies death by 2030 by scaling up their response to consign rabies to the history books [1]. Epidemiological surveys and phylogenetic analyses indicate elimination of rabies in wild animals and stray dogs are important for control of human rabies in China [4]. Further understanding on these issues would assist the development of measures such as better vaccines for control of human rabies [4, 9].

It is necessary to understand the local epidemiological characteristics of human rabies for providing a reliable epidemiology basis for further control and prevention of human rabies in Chongqing. Therefore, we carried out an epidemiological analysis of the human rabies surveillance data and the information on the post-exposure prophylaxis (PEP) of human rabies cases from 2007 to 2016, in order to improve the public health management strategies in Chongqing.

## Methods

### Information sources

The epidemiological data of human rabies in Chongqing from January 2007 to December 2016 were retrieved from the surveillance database of the “National Disease Reporting Information System” (NDRIS) of the Chinese Center for Disease Control and Prevention (CDC). According to the law of the People’s Republic of China on Prevention and Treatment of Infectious Diseases, all human rabies cases should be reported to NDRIS within 24 h after diagnosis. Human rabies is diagnosed according to the National Diagnostic Criteria for Rabies (WS 281–2008).

To investigate the situation of rabies exposure and the PEP of human rabies cases, a standardized questionnaire was administered face-to-face to 548 cases or their family members by trained investigators after informed and consent. The questionnaire covered four parts: (a) the cases’ demographic profile (name, gender, age, occupation); (b) exposure characteristics (date of event, site of lesion, the animal vector, the category of exposure); (c) PEP (wound washing, vaccination and/or immunoglobulin administration); and (d) clinical manifestation. The categories of exposure were classified according to the World Health Organization’s (WHO) guideline [10].

### Statistical analysis

Initial data were entered into EpiData software 3.1 and analyzed. Descriptive analyses were performed and presented in percentage or median and interquartile range to describe the demographic characteristics of the cases. The incubation time was assessed by Kruskal-Wallis H test or Kolmogorov-Smirnov Z test wherever appropriate. *P* < 0.05 was considered to be statistically significant.

## Results

### Epidemiological characteristics

**Fig. 1:**
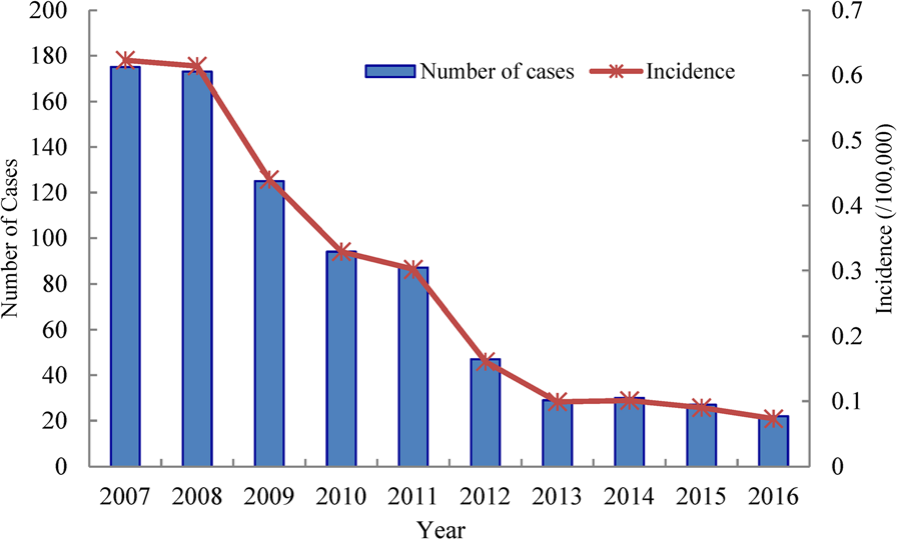
The annual number of cases and incidence rate for human rabies in Chongqing, China, 2007–2016. The blue columns indicate the number of human rabies cases and the red line indicates the incidence rate of human rabies cases

According to NDRIS, a total of 809 human rabies cases were reported and died in Chongqing from 2007 to 2016, the annual average incidence of the disease was 0.3 per 100,000 inhabitants per year. The annual incidences of human rabies showed a decreasing trend from 2008 to 2013, and then remained stable from 2013 to 2016. The annual number of cases and incidence rates for human rabies are summarized in Fig. 1.

**Fig. 2:**
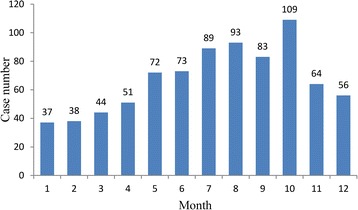
The human rabies cases by month in Chongqing, China, 2007–2016. The blue columns indicate the number of human rabies cases per month

Human rabies cases were reported throughout the year. However, 64.2% (519/809) of the cases occurred in summer or autumn seasons (from May to October) and the numbers reached peak in October, accounting for 13.5% of the total cases. The monthly distribution of human rabies cases are shown in Fig. 2 (see Additional file 1: Supporting file 1 for more details).

The age of human rabies cases ranged from 1 to 93, with a median of 53 years old. The peak ages of the cases fell in the 5–14 and 35–74 age groups, with 113 and 565 cases, respectively, which accounted for nearly 83.8% of the total cases. The male to female ratio was 1.9:1. The highest number of cases was found in farmers, which accounted for 71.8% of the total cases, followed by students or kindergarten-age children (15.4%). Overall, these two populations jointly accounted for 87.2% of the total cases. Demographic characteristics of human rabies cases from 2007 to 2016 in Chongqing were listed in Table 1.

**Table 1:** Characteristics of human rabies cases in Chongqing, China, 2007–2016

Characteristics	Number of human rabies cases	Proportion (%)
Age group		
0 to 4 years	33	4.1
5 to 9 years	57	7.1
10 to 14 years	56	6.9
15 to 24 years	27	3.3
25 to 34 years	27	3.3
35 to 44 years	99	12.2
45 to 54 years	122	15.1
55 to 64 years	230	28.4
65 to 74 years	114	14.1
75 to 93 years	44	5.5
Gender		
Male	528	65.3
Female	281	34.7
Occupation		
Farmer	581	71.8
Students or Kindergarten-age children^a^	125	15.5
Scattered children^b^	31	3.8
Housewife or Retiree	17	2.1
Worker	14	1.7
Medical staff	2	0.3
Others	39	4.8

^a^Students or Kindergarten-age children refer to those students studied in kindergarten, primary school, middle school or university

^b^Scattered children refer to children aged less than 3 years old

The top four districts or counties with largest numbers of human rabies in Chongqing were Wanzhou (54 cases), Hechuan (52 cases), Tongnan (49 cases) and Fuling (47 cases), and all of them are rural areas.

### Exposure and incubation

Exposure and PEP information were collected from 548 (67.7%) of all the reported rabies cases from 2007 to 2016. Of them, 525 (95.8%) cases had a history of dog bite or scratch and another 20 (3.6%) were attributed to cats (Table 2). Of the animal vectors, 292 (53.3%) were identified as stray animals and 253 (46.2%) were domestic dogs (belonging to the family of the case or a neighbor). Only 4.0% (10/253) of the domestic dogs had received rabies vaccine.

**Table 2:** Exposure characteristics of human rabies cases in Chongqing, 2007–2016

Exposure characteristics	No. of human rabies cases	Proportion (%)
Animal vector		
Canine	525	95.8
Cat	20	3.7
Wild animal	3	0.5
Animal source		
Stray dogs or cats	292	53.3
Domestic dogs	253	46.2
Wild	3	0.5
Exposure reasons		
Active attack by the vector	404	73.7
Defensive attack by the vector	55	10.0
Playing with the vector	69	12.6
Other	20	3.7
Exposure level^a^		
III	411	75.0
II	137	25.0
Exposure site		
Upper limb	251	45.8
Lower limb	173	31.6
Head/neck	95	17.3
Trunk	29	5.3
Incubation period		
≤ 30 days	150	29.0
30–60 days	131	25.3
61–90 days	95	18.4
91–120 days	53	10.3
121–150 days	20	3.9
151–180 days	15	2.9
181–365 days	40	7.7
> 1 year	13	2.5

^a^Category II exposure: nibbling of uncovered skin, minor abrasions or abrasions without bleeding. Category III exposure: single or multiple transdermal bites or scratches, licks on broken skin, contamination of mucous membrane with saliva from licks

According to the classification of contact with animals issued by WHO, 137 (25.0%) cases were classified into category II exposure (i.e., nibbling of uncovered skin, or minor abrasions without bleeding) and 411 (75.0%) were classified into category III exposure (i.e., transdermal bites or scratches, or mucous membrane contamination). The major wounds were occurred on the upper limbs (45.8%), followed by the lower limbs (31.6%), the remaining were on the head/ neck (17.3%) and trunk (5.3%).

According to the available data on the incubation period of 517 cases, the median was 54 days (ranged from 2 to 1476 days). The majority of cases occurred within 30 incubation days (29.0%), followed by the 30–60 days (25.3%). The incubation period of 72.7% cases was less than 3 months, while 13 cases (2.5%) had an incubation period over 1 year. Six cases (1.2%) had a short incubation period of less than 1 week after recognized exposure, while the longest reported incubation period in this study was 4 years, following a dog bite vaguely recalled by the case’s relative.

The incubation period was significantly shorter in cases with exposure sites on the head/neck (median = 22) than cases with a wound on trunk, upper or lower limbs (*p* < 0.01) (Table 3). Similarly, the incubation period was significantly shorter in cases with III exposure than in cases with II exposure (*p* = 0.031).

**Table 3:** The incubation periods among different exposure levels and sites of the human rabies cases in Chongqing, China, 2007–2016

Variables	Median (days)	Interquartile range (days)
Exposure site		
Head/neck	22	14, 50
Trunk	43	14.5, 92
Upper limb	55	31, 91
Lower limb	75	44, 145
Exposure level		
II	68	35, 115
III	50	25, 89

### Use of rabies post exposure prophylaxis (PEP)

Of the 548 cases with available PEP information, 290 cases (52.9%) had no wound treatment post exposure at all. Another 191 cases (34.9%) treated the wound by themselves, and only 67 cases (12.2%) went to the hospital for medical treatment (Table 4).

**Table 4:** Post-exposure prophylaxis of human rabies cases in Chongqing, 2007–2016

Variables	Number of human rabies cases	Proportion (%)
Wound treatment		
Untreated	290	52.9
Self-treated	191	34.9
Treatment in medical institution	67	12.2
Active immunization		
No	471	85.9
Incomplete full regimen	69	12.6
Complete full regimen	8	1.5
Passive immunization		
Yes	14^a^	2.6
No	534	97.4
Treatment time for PEP (days)^b^		
0	241	94.9
1–3	6	2.3
≥ 4	7	2.8

^a^The total 14 cases that received RIG comprised of 13 cases of category III exposure and 1 case of category II exposure

^b^The treatment time for PEP refers to the interval between potential exposure and receive PEP

With regard to the post-exposure vaccination, 69 cases (12.6%) received one to four doses of rabies vaccine, only 8 cases (1.5%) completed the full regimen of 5 doses, and the remaining cases did not receive any dose. Of the 8 cases who received the full regimen of 5 doses, 7 cases were category III exposure but did not received rabies immunoglobulin (RIG) which was recommended in WHO guidelines, and 6 cases did not receive the first shot of rabies vaccination on time. In summary, none of the 548 cases investigated in this study received adequate PEP according to the WHO guidelines. A total of 14 cases comprised of 13 with category III exposure and 1 with category II exposure received RIG.

## Discussion

A total of 809 human rabies cases were reported in Chongqing, China, from 2007 to 2016, all of them were dead. The number of human rabies cases gradually decreased from 2007 to 2013, which is in consistence with the epidemic trend of human rabies in China. This phenomenon may be due to rabies control being emphasized and strengthened by central government in China after 2007. The Treatment Guidelines for Rabies Post-exposure Prophylaxis of Humans were updated and applied then, which may attribute to the decline of human rabies cases. However, the human rabies cases remained stable from 2013 to 2016 (22–30 cases per year) in Chongqing, which clearly demonstrated that human rabies constituted a real public health issue in this area.

The major proportion of human rabies cases occurred in summer or autumn seasons, when more frequent outdoor activities with less cloth wearing increased the chance of rabid dog contact and exposure. Geographical distribution of human rabies indicated that most of the cases were in rural areas. The proportion of farmers in the cases was significantly higher than that in the total population [11] (see Additional file 1: Supporting file 2 for details). This phenomenon might be related to the chance of exposure, delay in obtaining PEP after potential rabies exposure (see Additional file 1: Supporting file 3 for details), lower proportion of cases that sought medical treatment (see Additional file 1: Supporting file 4 for details), and the long distance to health centers to receive PEP in rural areas [12].

It is noteworthy that besides adults aged 35–74 years old, children aged 5–14 years old also constituted the major cases. For fear of being scolded, children may not tell their guardians after bitten by animals; therefore they might not receive any treatment. Moreover, due to their height, they were more likely to have exposures on head/neck compared with adults [13] (see Additional file 1: Supporting file 5 for details). Similar to other studies [14–18], males were more frequently infected than females and the proportion of males in cases was significantly higher than that in the total population (see Additional file 1: Supporting file 2 for details); this phenomenon was possibly related to occupational or behavioral factors that making them more frequently contact with the animal vectors. Therefore, children and rural residents especially males aged 35–70 years could be the priority targets for rabies control and prevention in Chongqing, people should be more vigilant during summer and autumn periods to avoid contacting with animals.

The median incubation period was 54 days for human rabies cases in Chongqing, which consisted with the incubation period stated by the WHO (1–3 months). Similar to the results of other studies [19, 20], the incubation period was different for cases with different exposure sites and levels. Cases with a wound on the head/neck had the shortest incubation period compared to those with wounds on other sites; the incubation period of cases with category III exposure was significantly shorter than that of cases with category II exposure. The 6 special cases with incubation period less than 1 week were multiple transdermal bites on head/neck and didn’t receive any PEP. Those cases with an incubation period over 1 year suffered minor abrasions or abrasions without bleeding and were regarded as category II exposure. It is worth noting that 25% of the human rabies cases had a category II exposure in our study, which emphasized the importance of timely and appropriate PEP for exposure without bleeding.

Rabies is almost 100% fatal once clinical symptoms appear. Fortunately, it can be prevented by giving timely and appropriate PEP [21–23]. The WHO recommends immediate and thorough wound washing for a minimum of 15 min with soap and flowing water, full regimen of rabies vaccine and RIG for cases with category III exposure, which is considered as severe exposure. However, few cases sought medical treatment in our study, and none of them received adequate PEP according to the WHO guidelines. These results indicated the public’s ignorance of human rabies in Chongqing, which was more serious than in other areas [15, 19, 24]. The reasons that cases did not receive recommended PEP after exposure were not investigated in our study, it may be related to several factors, such as the prohibitive costs of vaccine and RIG, the lack of awareness about necessity of PEP and inadequate access to vaccine and RIG in primary health services especially in rural areas [25] (see Additional file 1: Supporting file 6 for details). Therefore, appropriate actions should be implemented and strengthened including (1) increase subsidies for rabies medication by expanding the scope of medical insurance for rabies PEP, just as some high-incidence human rabies provinces in China like Hunan and Guangdong, which have included rabies PEP expenditures into the new rural cooperative medical reimbursement coverage, (2) educate the public on the need of rapid treatment post exposure [26] and use education approaches such as short-message-service or WeChat, which might be useful tools for health promotion [27], (3) educate public health workers to evaluate and identify potential exposures to rabies and provide adequate PEP [28], (4) establish “animal bite treatment centers” in rural areas for better treatment and surveillance of rabies and animal bites in Chongqing.

Dogs play an important role in the transmission of human rabies infections in Chongqing, which is similar to other countries in Asia, Latin America, and Africa [12, 16, 19, 29, 30]. The exposure investigation in our study revealed that only 4.0% of the domestic dogs related to the cases were reported that have been vaccinated previously, which is far lower than the threshold of 70% vaccination coverage for efficient rabies control. Stray dogs were the principal animal responsible for the transmission of rabies in Chongqing. Therefore, mass implementation of canine vaccination of domestic dogs and effective measures to control stray dogs were the most logical solutions to reduce the burden of human rabies in Chongqing, rather than solely focusing on human PEP [31, 32].

Successful experiences of the elimination of human rabies in regions, such as Europe, Japan, Caribbean and Latin American provide a promising method for Chongqing to develop a more effective approach for controlling human rabies [33–35]. Such programs include implementing mass dog vaccination and management, canine rabies outbreak surveillance, health education to the general public especially high risk populations, implementing routine surveillance of rabies exposure in schools, and training the medical personnel to provide appropriate PEP for any potential exposure; these focused on the concept of “One Health” and required interdisciplinary collaboration from the public health and veterinary services, ministries of education, local authorities and non-governmental organizations [4, 36–39].

To our knowledge, this is the most comprehensive study showing the situation of human rabies in Chongqing, China. The data obtained from NDRIS may not well capture all the human rabies cases during this period in Chongqing because of the following reasons: some people in remote rural areas may not have easy access to medical treatment and clinical diagnosis, which lead to underestimated rabies incidence due to the confusion with other neurological infections [40, 41]. Thus, it is possible that the number of human rabies cases in this study were under-reported. Furthermore, some limitations of this study merit noting, including the lack of laboratory testing to confirm rabies, recall bias (particular for cases that involved a long incubation period or unconscious cases whose information recollections depended almost entirely on their family members or relatives) and incomplete information of PEP for several cases. Despite these limitations, this study provides useful information on the profile of human rabies in Chongqing, which could contribute to the control and prevention strategies for human rabies in this area.

## Conclusions

Human rabies remains a major public health problem in Chongqing, China. Unsuccessful control of canine rabies and inadequate PEP of cases might be the main factors leading to the serious human rabies epidemic in this area. It is crucial to implement a series of programs based on the concept of “One Health”, including implementing mass rabies vaccination, strict control of stray dogs, education of the public on the risk and prevention of rabies, implementing routine surveillance of rabies exposure in schools, and training medical personnel to provide appropriate PEP. As such, the elimination of human rabies in this area in the end will then be possible.

## Additional file

Additional file 1: Tables S1. The monthly distribution of human rabies cases in Chongqing from 2007 to 2016. **Table S2.** Comparison of the population distribution of human cases with that of the local population in Chongqing. **Table S3.** The interval of exposure and treatment time for PEP. **Table S4.** The proportion of cases that sought medical treatment. **Table S5.** The proportion of cases with exposure on head/neck. **Table S6.** PEP ambulant clinic conditions in Chongqing, 2016. (XLSX 20 kb)

## Abbreviations

AIDS: Acquired immunodeficiency syndrome
CDC: Center for disease control and prevention
NDRIS: National disease reporting information system
PEP: Post-exposure prophylaxis
RIG: Rabies immunoglobulin
USD: United States dollar
WHO: World Health Organization
